# Selective COX-2 Inhibitors as Neuroprotective Agents in Traumatic Brain Injury

**DOI:** 10.3390/biomedicines12081930

**Published:** 2024-08-22

**Authors:** Matthew I. Hiskens, Anthony G. Schneiders, Andrew S. Fenning

**Affiliations:** 1 Mackay Institute of Research and Innovation, Mackay Hospital and Health Service, Mackay, QLD 4740, Australia; 2 School of Health, Medical and Applied Sciences, Central Queensland University, Rockhampton, QLD 4701, Australiaa.fenning@cqu.edu.au (A.S.F.)

**Keywords:** traumatic brain injury, neuroprotection, neuroinflammation, inflammation, cyclooxygenase

## Abstract

Traumatic brain injury (TBI) is a significant contributor to mortality and morbidity in people, both young and old. There are currently no approved therapeutic interventions for TBI. Following TBI, cyclooxygenase (COX) enzymes generate prostaglandins and reactive oxygen species that perpetuate inflammation, with COX-1 and COX-2 isoforms providing differing responses. Selective COX-2 inhibitors have shown potential as neuroprotective agents. Results from animal models of TBI suggest potential treatment through the alleviation of secondary injury mechanisms involving neuroinflammation and neuronal cell death. Additionally, early clinical trials have shown that the use of celecoxib improves patient mortality and outcomes. This review aims to summarize the therapeutic effects of COX-2 inhibitors observed in TBI animal models, highlighting pertinent studies elucidating molecular pathways and expounding upon their mechanistic actions. We then investigated the current state of evidence for the utilization of COX-2 inhibitors for TBI patients.

## 1. Introduction

Traumatic brain injury (TBI) is a significant cause of disability and death worldwide [1]. It is projected that globally each year, sixty-nine million individuals sustain a TBI [2]. TBI survivors may suffer life-long consequences [3], with significant socio-economic consequences [4] and high medical costs resulting from TBI [5,6]. Overall, the most common TBI mechanisms are falls and traffic accidents [7,8]. TBI incidence is unequally distributed across different sexes and ages due to the varied environments and mechanisms of injury, with a higher proportion of males than females suffering TBIs [9]. In older women, falls are the primary cause of TBIs, whereas in young men, the leading causes are traffic accidents, violence, and sports injuries [10]. The age distribution of TBI exhibits a bimodal pattern, with peaks observed in individuals under 14 years and those over 65 years [11,12].

The diverse etiology, presentation, pathophysiology, complexity, and outcomes associated with TBI lead to challenges in patient care [13]. The multifaceted pathophysiology of TBI is initiated by a primary injury, which sets off a complex cascade of secondary injuries involving cerebral autoregulation impairment, leakage of the blood–brain barrier (BBB), formation of edema, oxidative stress, disruption of calcium homeostasis, and mitochondrial dysfunction [14,15,16,17]. These interrelated mechanisms result in neuronal cell death and prolonged inflammatory responses in the brain, which can lead to a range of clinical conditions including acute seizures, chronic epilepsy, neuroendocrine dysfunction, depressive disorders, and chronic traumatic encephalopathy (CTE) [18].

## 2. Current TBI Treatment Options

Despite being a leading cause of mortality, effective guidelines for the acute and long-term management of TBI remain elusive [19]. Acute therapeutic strategies for patients with moderate to severe TBI may include surgical procedures such as intracranial hematoma evacuation, decompressive craniectomy, and supportive interventions to alleviate symptoms and sustain homeostasis. Recent advancements in neuromonitoring, neuroimaging, and surgical techniques have contributed to improved outcomes and reduced mortality rates [20,21,22,23,24]. However, treatment trials or meta-analyses are complicated by varying and individualized treatment strategies that could be a contributor to the failure of TBI pharmaceutical interventions in clinical trials [25]. By targeting the acute injury-derived humoral sequalae with anti-inflammatory agents, the progression to more severe and chronic injury may be prevented.

## 3. Inflammation Following TBI

Inflammation is a fundamental component of TBI pathophysiology, irrespective of the injury’s severity [26,27]. More severe brain injuries evoke a more substantial and prolonged inflammatory response [28,29,30] but are also characterized by significant physical trauma. Neuroinflammation functions as an immune response to facilitate debris clearance and tissue repair. However, neuroinflammation has both beneficial and harmful effects on neuronal survival and brain repair, with excessive or prolonged inflammation exacerbating neuronal damage and contributing to neurological deficits [18,31,32,33]. Inflammatory responses also typically overshoot their physiological requirements, which promotes excessive tissue damage and remodeling [30].

**Figure 1:**
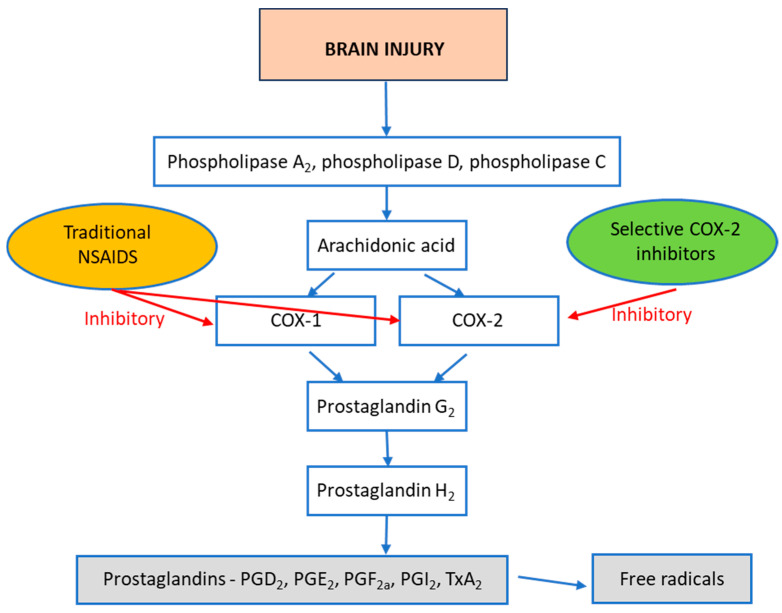
Generation of prostaglandins and free radicals following TBI. TBI increases the activity of phospholipase A_2_, phospholipase D, and phospholipase C enzymes, resulting in arachidonic acid generation. COX-1 and COX-2 enzymes convert arachidonic acid into prostaglandin G_2,_ which is then converted into prostaglandin H_2_. Prostaglandin H_2_ is then converted into prostaglandin analogs and thromboxane A_2_. These metabolites trigger physiological effects, and also result in the further generation of free radicals.

Inflammation following TBI triggers the phosphorylation of phospholipase A_2_, phospholipase D, and phospholipase C, which release arachidonic acid (Figure 1). The cyclooxygenase (COX) enzymes, COX-1 and COX-2, transform arachidonic acid into prostaglandin G2 (PGG_2_). This is then further metabolized into prostaglandin H2 (PGH_2_) with the aid of the peroxidase enzyme. Subsequently, PGH_2_ is transformed into various prostaglandin molecules, such as PGD_2_, PGE_2_, PGF_2_, and PGI_2_ (prostacyclin), and thromboxane A2 (TxA_2_). These resulting prostaglandins and related compounds are propagators of inflammation and contribute to a wide range of inflammation signaling processes involved in secondary injury mechanisms [34].

Inflammation is also intrinsically linked with oxidative stress pathways. Elevated levels of intracellular calcium ions can result in cell edema and a decrease in cerebral blood flow [35]. This damages mitochondria and leads to the increased generation of free radicals. These free radicals damage cellular membranes through lipid peroxidation, which results in an increase in arachidonic acid in the cytosol. This contributes to arachidonic acid’s production of thromboxane and prostaglandin metabolites, which themselves generate more free radicals.

## 4. Anti-Inflammatory Drugs for TBI

A complete review of the current state of anti-inflammatory pharmacological interventions for TBI is beyond the scope of this paper, but has recently been performed [25]. One key strategy to reduce neuroinflammation involves reducing the production of prostaglandins by administering glucocorticoids or COX inhibitors. These compounds also decrease the production of free radicals, thromboxanes, and prostacyclins [36].

Glucocorticoids block COX-2 expression without affecting COX-1 expression. However, glucocorticoids may have significant adverse effects [37] and their efficacy in reducing inflammation following TBI may be limited [38]. The glucocorticoids methylprednisolone and dexamethasone have been investigated as potential TBI treatments. Early dexamethasone administration has shown promise in reducing edema through aquaporin-1 regulation [39] and microglial inhibition [40]. However, corticosteroid therapies have failed to demonstrate efficacy in clinical trials. This was exemplified by the termination of the MRC CRASH trial, which observed an elevated mortality rate in the 14 days post-TBI among patients administered methylprednisolone [37]. As a result of this evidence, corticosteroids are not being investigated as a TBI treatment [41].

**Figure 2:**
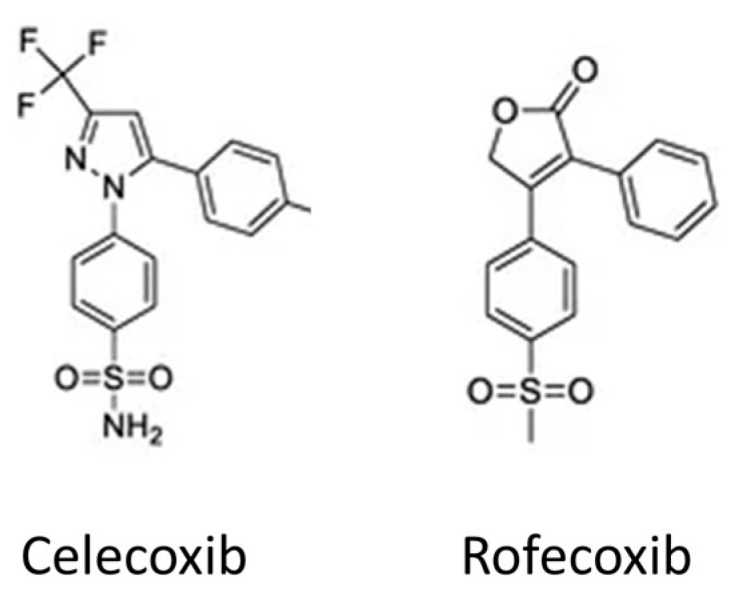
Structure of celecoxib and rofecoxib.

Non-steroidal anti-inflammatory drugs (NSAIDs) modulate the inflammatory response by targeting COX enzyme pathways activated in response to trauma by reducing (i) prostaglandins and thromboxanes, (ii) cytokines, and (iii) proteases [42]. COX enzymes are present in two distinct isoforms, namely, COX-1 and COX-2. COX-1 is ubiquitously expressed across various tissues, indicating its non-specific distribution. However, the expression of COX-2 is specifically triggered by inflammatory products, growth factors, and hormones [43]. First- and second-generation NSAIDs were developed to non-selectively inhibit both COX-1 and COX-2 enzymes. However, a notable side effect of COX-1 inhibition is the upregulation of gastric acid production, with adverse effects including gastric ulceration, bleeding, and renal dysfunction [44,45]. In contrast, it was identified that COX-2 inhibition elicits anti-inflammatory effects without these drawbacks. Therefore, the third generation of NSAIDs were designed for selective COX-2 inhibition. Two selective COX-2 inhibitors, celecoxib and rofecoxib (Figure 2), became available on the international market in 1999. These compounds were approved for conditions such as rheumatoid arthritis and osteoarthritis, while simultaneously demonstrating a significant reduction in hemorrhagic GI complications. However, as the use of rofecoxib increased, data emerged from several studies suggesting an increased risk of cardiovascular events such as myocardial infarction and stroke. This led the FDA to instruct Merck to include rofecoxib packaging with precautionary labels. Further studies of rofecoxib suggesting cardiovascular hazards led to its withdrawal from the market, such that celecoxib is now the only selective COX-2 drug available in the United States. It has been demonstrated that celecoxib at moderate doses shows a similar risk of cardiovascular events as non-selective NSAIDs [46]. Celecoxib is thought to be associated with a lower risk of cardiovascular events than rofecoxib because it is a less selective COX-2 inhibitor.

The rationale for COX enzymes as a therapeutic strategy for addressing TBI neuroinflammation stems from studies revealing NSAID protection in Alzheimer’s disease (AD) patients [47,48]. COX inhibition exhibits anticipated analgesic and anti-inflammatory effects, with emerging evidence suggesting neuroprotective properties in conditions involving neurotoxicity, neurodegeneration, and demyelination [49,50,51,52,53]. In an AD model, celecoxib administration led to reduced astrocyte and microglia upregulation, prevented behavioral impairment, and normalized neurotransmitter response [54]. Other evidence of therapeutic potential has also been demonstrated in preclinical models of neurodegeneration, where celecoxib treatment has protected against learning and memory impairments [55]. Notably, neuroprotection is not solely attributed to inflammation pathways, as COX-2 inhibition has been found to invoke increased neuronal survival in the absence of decreased mRNA inflammatory signaling [56].

Selective COX-2 inhibitors are posited to enhance patient outcomes in TBI cases by mitigating secondary injury related to inflammation, free radicals, and edema [57,58]. However, the role of COX-2 inhibitors in TBI presents a paradox; reservations primarily revolve around the uncertainties with administration timing and dosage, and the potential repercussions of negating protective repair and recovery processes [59]. While excellent reviews of COX-2 inhibitor treatment in TBI have been undertaken previously [60,61], we aim to provide an update on the state of the literature, and to our knowledge, this is the first review of COX-2 inhibitors for TBI treatment that includes both animal and human studies. The following sections explore the preclinical and clinical evidence for the role of COX-2 inhibition in TBI.

## 5. Preclinical Studies

### 5.1. Injury Administration and Assessment

Understanding the experimental methods applied in TBI models is important for interpreting the outcomes, as a diverse range of TBI models have been employed to evaluate experimental therapeutics. TBI animal models are broadly categorized into closed head injury models, where the skull is not surgically altered, and open head injury models, where TBI is induced through a craniotomy [62]. Among closed head models, a weight drop on the skull is a common method, producing a focal injury to the ipsilateral cortex and hippocampus [62]. These weight drop methods can be further sub-categorized; for example the Marmarou weight drop model is a common method involving a helmet affixed to the rodent’s head in order to produce a diffuse TBI. Open head injury models, such as lateral fluid percussion (LFP) and controlled cortical impact (CCI) yield focal injuries in the cortex, with more severe injuries potentially damaging the underlying hippocampus. The uniformity and consistency of injury can vary based on the method and the use of implements such as a stereotaxic frame.

It is important to highlight that no single injury model can replicate the wide spectrum of injury biomechanics seen in human TBI. However, preclinical models can reproduce and elucidate details of relevant pathophysiology observed in human TBI patients. This provides the opportunity to standardize preclinical studies in order to prevent the heterogeneity of etiology and clinical complexity in human TBI. For these reasons, the majority of TBI research is undertaken in animal models [63,64].

Assessing neurological outcomes to evaluate therapeutic efficacy is undertaken by testing reflexes or motor function, signs of anxiety or depression, and cognitive function or memory [27]. However, it is essential to recognize that most models induce transient impairment of reflexes and motor function that spontaneously recover in the days following TBI. Consequently, assessing the effectiveness of a drug through the measurement of reflexes or motor impairments carries the implication that it is the rate of recovery that is being evaluated. Unlike deficits in reflexes and motor functions, impairments in cognitive and memory faculties can be permanent and provide opportunities to measure the neuroprotective potential of a drug. Models of TBI elicit measurable anxiety or depression-like responses in animals that mirror the alterations in emotional state that are frequently observed following clinical TBI. Tests of cognition or affect involve the animal moving around a maze or apparatus, making it necessary to couple the cognition test with a test confirming the absence of motor deficits to avoid false-positive cognitive deficit results.

### 5.2. Findings in Preclinical Studies of Selective COX-2 Inhibitors

Investigations of the modulation of TBI sequelae has targeted several categories of agents, with a collection of experimental studies examining the effects of selective COX-2 inhibitors. These COX-2-selective drugs have included carprofen, celecoxib, diclogenac, DFU, meloxicam, nimesulide, and roficoxib, across various animal models of TBI. These agents have been administered either prior to injury or within a 30 min post-injury timeframe. Table 1 displays the details of these investigations and the pathological and functional outcomes.

### 5.3. Pathology

A diverse range of pathological outcomes has been investigated to assess the efficacy of these COX-2-selective inhibitors. Protein expression of COX-2 has been measured in a number of studies, with differing results. When injected directly into the site of focal penetrating TBI, diclofenac exhibited no reduction in COX-2 [69], and rofecoxib also had no impact on COX-2 expression [73]. In contrast, DFU treatment decreased COX-2 levels when administered 10 min prior to trauma, but not when administered 2–6 h after TBI [70].

Several measures of inflammation have been investigated. Microglial activation as measured by Iba-1 expression has been shown to be reduced at 4 h post-injury with carprofen [65] and 48 h post-injury with celecoxib treatment [67]. Carprofen and celecoxib also exhibit a notable inhibition of the proinflammatory cytokine IL-1β [65,68]. However, there were no differences in the brain IL-10 levels after TBI with carprofen or celecoxib treatment. DFU, meloxicam, and nimesulide effectively reduced prostaglandin production [35,65,66,70,71]. By reducing prostaglandin production following TBI, arachidonic acid metabolism may be directed toward a neuroprotective eicosanoid pathway.

Several studies examined the effects of treatment on tissue injury. Diclofenac-mediated COX-2 inhibition decreased apoptosis in injured rats, while modulating secondary injury mechanisms involving microglial cells and astrocytes [69]. Gopez et al. also showed that DFU was effective in reducing cell death [70]. Carprofen treatment resulted in decreased lesion size in a weight drop model of TBI [65]. Rolicoxib, when administered 5 min post-injury, did not show an effect upon hippocampal neuronal loss [73]. Nimesulide exhibited no discernible impact on edema, and the effect of meloxicam on edema remains inconclusive, with Hakan et al. [35] observing a reduction in TBI-related edema after meloxicam administration, contrasting with Girgis et al.’s findings of no change in edema with meloxicam treatment [35,71]. Hakan and colleagues proposed that the observed neuroprotective benefits of meloxicam could be partially attributed to its antioxidant characteristics, in addition to the anti-inflammatory effects of COX-2 inhibition. In this way, it could be that COX-2 inhibitors contribute to neuroprotection by curbing the excessive production of free radicals following TBI.

### 5.4. Functional Outcomes

There is significant variability in the performance of these selective COX-2 inhibitors in functional outcomes involving motor function. Reflexes and motor function were primarily assessed via the neurological severity score (NSS), which was improved following TBI with administration of carprofen and DFU [65,70]. There were discrepant NSS findings with meloxicam, with improvement in the study by Hakan and colleagues and no change in the study by Girgis et al. [35,71]. Nimesulide treatment effected no change in NSS; however, there was improved rotarod performance [71,72]. DFU treatment resulted in no change in beam walk function [70], while Dash et al. revealed a worsening of motor function in rats post-TBI with celecoxib [66].

Cognitive function and memory were assessed using the Morris water maze (MWM) and the Barnes maze. Cognitive performance following celecoxib treatment did not improve MWM performance in the study by Dash et al. [66] and impaired MWM performance following repetitive weight drop mTBI [67]. Gopez et al. found that DFU improved MWM performance, while nimesulide treatment improved Barnes maze performance [70,72].

### 5.5. Evaluation of Preclinical Studies

Two notable concepts emerge from the findings of these preclinical TBI studies: (1) the wide range of effectiveness of the different agents across pathological and functional outcomes, and (2) the disconnect between improvements in histological neuronal damage and the lack of functional improvement, highlighting the complexity of assessing therapeutic outcomes [75,76]. The discrepancies in these animal model findings could be multifactorial. One aspect could be the role of the age of the animals, which was shown by Hickey and colleagues to play a significant role in the expression of COX-2 following injury [74]. Neonatal and 90-day-old mice showed less TBI-induced COX-2 expression than mice between the ages of 14 and 60 days [74]. This varied COX-2 expression will therefore impact the effectiveness of pharmacological intervention. Other significant factors involve the variation in the methodology of drug administration, encompassing differences in route, dosage, and timing of delivery. Another significant source of variation is the differing mechanisms and sequelae of TBI, thus contributing to differences in TBI biomechanics, severity, and outcomes, further influencing the results.

Despite the often positive effect that selective COX-2 inhibitors enact upon cellular and histological measures of injury, these have failed to yield improvements in clinical and functional outcomes. This suggests that the anti-inflammatory effect, targeted at mitigating damage induced by TBI, proves ineffective in preventing damage across secondary injury cascades, ultimately leading to functional impairment. However, our understanding of the cascade of interaction resulting from treatment with these agents, and their exact mechanisms of actions, remains limited. For example, in the diclofenac treatment study, while COX-2 expression increased at the injury site, the effects of the drug were not only attributed to COX-2 inhibition. Instead, they involved the indirect modulation of inflammatory responses in astrocytes and microglial cells [69]. This suggests a strategy for targeting TBI through selective COX-2 inhibitors, even when not specifically directing treatment at COX-2 pathways. A more complete understanding of these interactions is required for designing COX-2 inhibition studies as an effective treatment of TBI and for enacting clinical improvement.

### 5.6. The Role of COX-1 in Brain Injury

While COX-2 is the predominant isoform in the brain, COX-1 may also play a role in brain neuroinflammation. Data from human TBI reveal the upregulation of COX-1+ microglia and macrophages in perilesional areas affected by the acute inflammatory response to TBI [77]. This is supported by preclinical data indicating COX-1 upregulation at the site of injury [78]. This indicates that COX-1 expression in the brain exhibits distinctions from its constitutive expression in peripheral tissues.

Pharmacological inhibition of COX-1 has been investigated in a rat model of TBI via treatment by the selective COX-1 inhibitor SC560, resulting in improvements in motor, spatial learning, and memory tasks [79]. These findings imply a role for COX-1 in the cognitive deficits associated with TBI. Additionally, the dual inhibition of COX-1 and COX-2 has been investigated in the context of TBI [71,80]. The non-selective COX inhibitors indomethacin and ibuprofen were administered prophylactically or within 10 min of injury and demonstrated a robust anti-inflammatory effect by inhibiting key mediators such as IL-1β, IL-6, IL-10, and prostaglandins in both serum and brain tissue [68,71,80]. However, indomethacin and ibuprofen showed a limited ability to improve other outcomes such as edema [71,80].

In understanding the comparative effects of COX-1 and COX-2, the study by Girgis et al. (2013) gives insight into the selective and non-selective effects of COX enzymes. While treatment with meloxicam and nimesulide did not reduce neurological impairment following TBI, the non-selective COX inhibitor indomethacin significantly improved neurological outcomes [71].

Collectively, these studies illustrate the anti-inflammatory effect of NSAIDs in experimental TBI. However, this anti-inflammatory effect, while robust, appears insufficient to completely mitigate tissue injury and resultant functional deficits. Consequently, these investigations challenge the viability of targeting COX1 or COX2 as a standalone and effective therapeutic approach for TBI.

## 6. Human Studies

To date, there have been limited clinical investigations of the treatment of TBI with selective COX-2 inhibitors. Indeed, a systematic search of the US National Institutes of Health clinical trials database using the keywords traumatic brain injury, TBI, cyclooxygenase, COX-2, and the individual drug names carprofen, celecoxib, diclogenac, DFU, meloxicam, nimesulide, and roficoxib revealed no current registered clinical trials.

In a small human trial, the COX-2 inhibitor SC-58125 was shown to reduce excitation-induced neuroinflammatory damage following moderate brain injury [81]. Twice-daily COX-2 inhibition lowered blood glutamate levels which were increased following a moderate TBI [81]. However, this investigation did not evaluate clinical outcomes.

Recently, a retrospective cohort study investigated the outcomes of TBI patients treated with the COX-2-inhibiting agent celecoxib and the non-specific COX inhibitor ibuprofen [82]. The study interrogated a United States healthcare database to analyze data from 1443 patients over the age of 18. The investigation revealed that patients receiving celecoxib within five days post-TBI exhibited a heightened 1-year survival probability in comparison to their untreated counterparts. Additionally, the celecoxib-treated cohort demonstrated a reduced likelihood of gastrostomy tube dependence, myocardial infarction, and seizures. The study also demonstrated that ibuprofen use within five days of TBI was associated with a higher 1-year survival probability and lower complication rates related to craniotomy/craniectomy, seizure, deep vein thrombosis, and ischemic stroke.

This first population-level study of acute COX inhibition in TBI provides important data on the corresponding clinical outcomes. In translating preclinical mechanistic findings, one can hypothesize that the observed findings of celecoxib treatment with reduced seizure incidence and enhanced 1-year survival probability may be attributed to a potential modulation of neuroinflammation. The details of how the effects of COX-2 inhibitors are specifically linked to pathways such as the downregulation of proinflammatory cytokines and attenuation of edema will be of interest in future prospective studies.

## 7. Discussion and Future Perspectives

The animal model and clinical studies undertaken to date provide a complex landscape in which to interpret the efficacy of COX-2 inhibition in TBI. The preclinical studies suggest COX-2 inhibitors provide effective treatment at a cellular and histological level, but this does not readily translate into improved functional outcomes. However, human data show improvements in key metrics of survival and comorbid outcomes. In the preclinical setting, the effectiveness of COX-2 inhibition is heavily influenced by the model of TBI, gender, and age.

Following TBI, the increase in arachidonic acid leads to the enhanced expression of COX-2, resultant prostaglandin and thromboxane production, and ultimately, the induction of inflammatory cytokines such as TNF, IL-1ß, and IL-6. The expression of these inflammatory cytokines in the brain contributes to physiological consequences such as cerebral edema, increased intracranial pressure, and neuronal dysfunction [65]. By inhibiting COX-2, celecoxib and other COX-2 inhibitors likely disrupt this pathogenic cascade, thereby offering therapeutic benefits. The studies in this review suggest evidence of this related to several mechanisms. For example, celecoxib attenuates injury-induced prostaglandin production in the brain, which may result in the inhibition of the proinflammatory cytokine TNF, which has been demonstrated to effect blood–brain barrier integrity, edema formation, and hippocampal neuronal loss following TBI [65]. Likewise, IL-1ß mediates inflammatory responses after TBI. Thus, COX-2 inhibitors such as celecoxib, which significantly decreased brain IL1- β levels, may exert some of their effects through this mechanism.

In exploring the preclinical data, the initial inflammatory response post-injury serves a protective role and altering COX-2 release in response to injury may inadvertently lead to deleterious consequences. For instance, the rapid elevation of brain prostaglandin levels following injury, accompanied by a reflective increase in thromboxane, is thought to mitigate the risk of brain hemorrhage [70]. Additionally, blocking PGE_2_ production with COX-2 inhibition has been shown to exacerbate the neuroinflammatory response [83], raising concerns that COX-2 inhibition may, in certain instances, induce detriment rather than benefit. Consequently, the introduction of COX-2 inhibitors may disrupt some adaptive processes, potentially leading to an acute exacerbation of secondary damage processes.

The early preclinical COX-2 treatment studies used pan-microglial markers such as Iba-1, meaning that the investigation could not differentiate between M1 and M2 microglial subsets [84,85]. It is noteworthy that the microglial markers utilized in these studies do not discriminate between resident microglia and infiltrating macrophages post-injury. Consequently, the conclusions drawn from studies exclusively using pan-microglial markers may not definitively establish whether a therapeutic agent exerts a proinflammatory or anti-inflammatory effect on microglia [84,86]. On the other hand, some studies measured specific inflammatory mediators associated with the M1 (iNOS) or M2 (IL-10) microglial phenotypes but did not elaborate on how the drug modulates overall microglial activation. Future examinations of microglial subsets should provide mechanistic evidence of how these COX-2 inhibitors exert their effects in limiting TBI pathology.

The time-sensitive nature of inflammation initiation after TBI necessitates immediate intervention in mitigating brain damage. Prophylactic treatment was used in some preclinical studies as an approach to mitigate this, leveraging the well-established safety profile of COX-2 inhibitors, while other preclinical studies used immediate post-injury timing. In clinical situations, administering the drug post-injury poses challenges in achieving initiation within the narrow post-injury window, and missing this critical timeframe significantly diminishes treatment efficacy [58]. Understanding the parameters for effective timing within the therapeutic window of these agents should be a key focus of future prospective studies. Additionally, other essential pharmacological parameters should be examined including dosage route, number, and timing. These details will refine our understanding of the diverse pharmacological aspects of treatment and allow for the translation of promising preclinical findings into clinically effective interventions.

Gender plays a role in the inflammatory response to TBI, with females better protected than males in experimental models, although the reasons for this are not fully known [87]. Likewise, COX-2 regulation exhibits gender-specific differences in TBI. Specifically, in a rat CCI model, males showed increased COX-2 expression, which correlated with elevated apoptotic cell death [88]. The gender-specific aspect of the secondary inflammatory response may be linked to prostaglandin regulation, potentially contributing to gender-related outcome disparities following TBI. Thus, the influence of gender on COX-2 pathways and injury is an important consideration for understanding the effectiveness of pharmacological intervention.

The safety aspect of COX-2 inhibitors is an important consideration for use. While celecoxib is a widely used agent with a well-established profile, the use of COX-2 inhibitors has been linked with adverse cardiovascular events for patients with increased risk factors [82]. Therefore, for patients who have known cardiovascular disease and elderly patients, celecoxib may be contraindicated for TBI treatment due to the higher risk of myocardial infarction. This risk is likely related to the inhibition of endothelial COX-2-derived prostacyclin but not platelet COX-1-derived TxA_2_. Interestingly, the clinical study by Bhanja and colleagues revealed an association between celecoxib use and lower rates of myocardial infarction [82]. These authors postulated that patients with known cardiovascular risk were treated more conservatively with COX2-inhibiting agents, or perhaps that celecoxib use might regulate TBI-induced coagulopathy. This risk paradox should be investigated in future treatment studies.

While not addressed in the preclinical or clinical work performed to date, the long-term effects of COX-2 inhibitor treatment for TBI will be an important consideration as a risk management tool for the development of the neurodegenerative condition CTE. While large-scale clinical trials of COX-2 inhibitors have not shown clear benefits in AD [89,90], these investigations have recruited patients with advanced disease, characterized by the presence of neurofibrillary tangles and neuritic plaques. The cellular inflammation pathways shared between AD and CTE highlight the necessity for the investigation of COX-derived CTE therapeutics, leveraging insights gained from extensive studies utilizing COX blockade in AD models [91]. An avenue for future investigation will involve exploring the potential of quenching early inflammatory cascades as a strategy for long-term protection against neurodegenerative conditions.

## 8. Conclusions

COX-2 inhibitors play a significant role in the context of TBI due to the role of COX-2 in producing prostaglandin metabolites and ROS that can exacerbate brain injury. The use of COX-2 inhibitors has been shown to attenuate neuropathology in animal models but is inconsistent in its ability to improve cognitive functions and motor performance. Additionally, the exact function of COX-2 in post-traumatic neuroinflammation remains unclear. In clinical use, the COX-2 inhibitor celecoxib has demonstrated improved survival for TBI patients. Given the complex but promising findings, future prospective studies are important to understand the therapeutic potential of COX-2 inhibitor treatment.

## Figures and Tables

**Table 1:** Selective COX-2 treatment in rodent models of TBI.

Drug	Animal	Mechanism	Dosage Timeframe	Inflammation	Pathology	Neurological Outcome	Reference
Carprofen	Mouse	WD	5 min post	⇓ Iba-1 ⇓ Il-1β ⇨ IL-4 ⇓ IL-6 ⇨ IL-10	⇓ Edema ⇑ Gliogenesis ⇓ Lesion area	⇓ NSS	Thau-Zuchman et al. (2012) [65]
Celecoxib	Rat	CCI	Pre-injury	-	-	⇓ Motor ⇨ MWM ⇨ Conditioned emotional response	Dash et al. (2000) [66]
Celecoxib	Mouse	M-WD	Pre-injury	⇓ Gfap ⇓ Iba-1 ⇓ TNF	⇓ MAPT	⇓ MWM	Hiskens et al. (2021) [67]
Celecoxib	Rat	WD	Pre-injury	⇓ Il-1β ⇨ IL-10	-	-	Khaksari et al. (2012) [68]
Diclofenac	Rat	CCI	Immediate	-	⇓ Apoptosis ⇓ Lesion area ⇨ Neuronal degeneration	-	Dehlaghi et al. (2019) [69]
DFU	Rat	CCI	10 min pre or 6 h post	-	⇓ PGE_2_ ⇓ Caspase-3 ⇑ 2-AG	⇓ Neuro score ⇨ Beam walk ⇨ Open-field ⇓ MWM	Gopez et al. (2005) [70]
Meloxicam	Rat	M-WD	30 min post	⇓ Lipid peroxidation ⇓ GSH ⇨ Na K ATPase	⇓ Edema ⇓ BBB	⇓ NSS	Hakan et al. (2010) [35]
Meloxicam	Mouse	WD	10 min post	-	⇓ 6-keto PGF_1α_ ⇨ Edema	⇨ Neuro score	Girgis et al. (2013) [71]
Nimesulide	Rat	M-WD	30 min post	-	-	⇓ Barnes maze ⇓ Rotarod	Cernak et al. (2001) [72]
Nimesulide	Mouse	WD	10 min post	-	⇓ 6-keto PGF_1α_ ⇨ Edema	⇨ Neuro score	Grigis et al. (2013) [71]
Roficoxib	Rat	LFP	5 min post	-	⇨ Neuronal degeneration	-	Kunz et al. (2006) [73]
SC58125	Rat	CCI	15 min post or 24 h post		⇓ PGE_2_ ⇨ Lesion area	⇑ MWM	Hickey et al. (2007) [74]

Abbreviations: ⇑, increased outcome measure; ⇓, decreased outcome measure; ⇨, no change in the outcome measure; 2-AG, 2-arachidonoyl glycerol; BBB, blood–brain barrier; CCI, controlled cortical impact; Gfap, glial fibrillary acidic protein; IL, Interleukin; IL-1β, Interleukin 1 beta; Iba-1, Ionized calcium-binding adapter molecule 1; LFP, lateral fluid percussion; MAPT, microtubule-associated protein tau; M-WD, Marmarou weight drop; MWM, Morris water maze; NSS, neurological severity score; PGE_2_, prostaglandin E2; TNF, tumor necrosis factor; WD, weight drop.

## Data Availability

Not applicable.
